# Supplementary material for: Ultrafast relaxation of photoexcited superfluid He nanodroplets
Source: Nat Commun. 2020 Jan 8;11:112. doi: 10.1038/s41467-019-13681-6 (PMC6949273; doi:10.1038/s41467-019-13681-6)
Supplement: Supplementary file 1 — Supplementary Information [file 41467_2019_13681_MOESM1_ESM.pdf]

Supplementary Information
Ultrafast relaxation of photoexcited superfluid He nanodroplets

M. Mudrich *et al.*

Supplementary Note 1: Systematics of He nanodroplet relaxation

Besides characterizing the relaxation dynamics of excited He nanodroplets as a function of the photon energy of the XUV pulse, $h\nu$, the mean He droplet size $\bar{N}$ was systematically varied by changing the temperature of the He nozzle in the range 10-26 K. Supplementary Fig. 1 shows the delay-dependent PES recorded at $h\nu = 22.2$ eV for three values of $\bar{N}$.

Surprisingly, the relaxation dynamics only slightly changes when varying $\bar{N}$ from 2×10^3 up to 3×10^8; the exponential time constant for the appearance of peak A derived from the fits of the PES increases from $\tau_{\mathrm{A}} = 0.4$ to 0.9 ps. This matches the theoretical finding that the bubble expansion in the bulk of the droplet, which is the more likely case for large droplets, takes a few 100 fs longer than when He* is close to the surface (Fig. 4 in the main text). More notably, the fraction of He* atoms inside He bubbles concomitantly rises from 0.6 to 5 by nearly a factor 10. This is because for larger $\bar{N}$, He* has a lower chance to leave the droplet in finite time and therefore the fraction of $1s2s\,^1S$ He* remaining inside a bubble (peak D at 0.9 eV) with respect to those ejected (peak A at 0.8 eV) increases. The broadening of peak A from 0.13 eV (FWHM), limited by the resolution of our VMI spectrometer, to 0.25 eV for large droplets is presumably due to scattering of the emitted electrons at the droplets [1].

When tuning the XUV laser within the $1s2p\,^1P$ band from $h\nu$ =21.3 eV to 22.2 eV at constant droplet size $\bar{N} = 5 \times 10^5$ (not shown), we find from the fits an increase of $\tau_{\mathrm{A}} = 0.5$ to 0.8 ps and a rise of the fraction of bubble-bound He* (peak D) from 0.9 to 4.5. Since the broadening of the droplet excited states is partly related to the inhomogeneous He density in the surface region of the droplets [2], the location of the He* with respect to the droplet surface can be controlled by $h\nu$ to some extent. Therefore, larger blue detuning from the atomic line results in excitation of He* deeper inside the droplet, which explains the experimental finding.

In addition to recording PES, time-resolved ion mass spectra were measured using a time-of-flight detector placed opposite to the electron VMI detector.

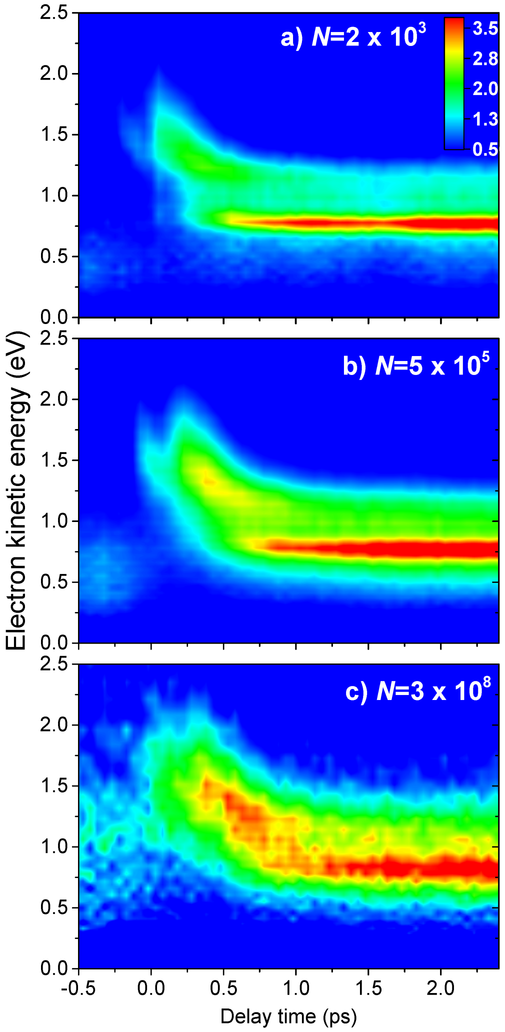

Supplementary Fig. 1: Time-resolved photoelectron spectra. The mean size of the He nanodroplets is varied as indicated in the legends. The photon energy of the pump pulse is held fixed at $h\nu = 22.2$ eV.

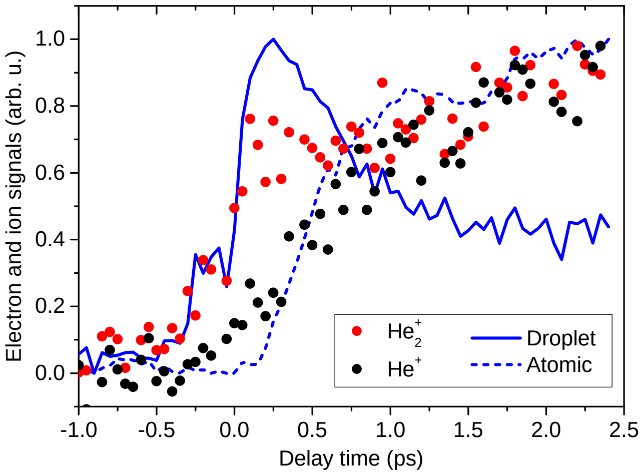

Supplementary Fig. 2: Evolution of the number of He^+ and He_2^+ ions. For comparison, the evolution of atomic component (peak A) and the droplet component (peak D) in the photoelectron spectra are shown as dashed and solid lines, respectively.

The delay dependence of the total counts of He^+ and He_2^+ ions is depicted in Supplementary Fig. 2 in comparison with the areas of the atomic and droplet peaks A and D, respectively. Both electron and ion spectra were recorded for He droplets of size $N = 2 \times 10^3$ at $h\nu = 22.2$ eV. Ion counts recorded at different values of N and $h\nu$ show very similar evolutions as in Supplementary Fig. 2. The main finding is that the He^+ signal rises at about 0.5 ps later delays than the He_2^+ signal, closely following the atomic peak A in the electron spectra, whereas the He_2^+ ion signal follows electron feature D. This result confirms our interpretation of feature D as representing the He^* excitation located inside the He droplet; when a He atom is photoionized in the vicinity of another He atoms it forms a vibrationally excited He_2^+ dimer, which eventually desorbs off the He droplet in the course of vibrational relaxation [3, 4]. In contrast, the He^+ ions originate from free He^* ejected out of the droplets by the decaying bubble. In previous photoionization experiments, free He^+ ions have been found to originate exclusively from either free He atoms which accompany the droplet beam [5], or from He atoms or complexes ejected out of the droplets in the ionization process [6, 7].

Supplementary Note 2: TD-DFT simulation

The density functional theory (DFT) and time-dependent density functional theory (TD-DFT) approaches to superfluid He are thoroughly described in Ref. [8]. Here, we give additional details about the initialization and propagation of the droplet-He^* system.

Starting from the equilibrium atom density of the $^4\text{He}_{N+1}$ droplet, we choose a point in space $\mathbf{r}_X$ at a distance d below the He droplet surface, where the excited He* atom is located. We construct the density

$$\rho_X(\mathbf{r}) \equiv \rho(\mathbf{r})\, g(|\mathbf{r}_X - \mathbf{r})|), \tag{1}$$

where $g(r)$ is the pair distribution function in liquid He as obtained from diffusion Monte Carlo calculations [9], normalizing $\rho_X(\mathbf{r})$ to N atoms. This procedure yields an "atom bubble" located at $\mathbf{r}_X$. We obtain the wave function $\Phi_X(\mathbf{r})$ for the He atom singled out at $\mathbf{r}_X$ that is excited to He*, and the droplet density $\rho(\mathbf{r})$, by self-consistently solving the static DFT and Schrödinger equations

$$\left\{-\frac{\hbar^2}{2m_{\text{He}}}\nabla^2 + \frac{\delta\mathcal{E}}{\delta\rho} + \int d\mathbf{r}' \mathcal{V}_{\text{He}-\text{He}}(|\mathbf{r}-\mathbf{r}'|)|\Phi_X(\mathbf{r}')|^2\right\}\Psi(\mathbf{r}) = \mu\Psi(\mathbf{r}) \tag{2}$$

$$\left\{-\frac{\hbar^2}{2m_{\text{He}}}\nabla^2 + \int d\mathbf{r}'\, \mathcal{V}_{\text{He}-\text{He}}(|\mathbf{r}-\mathbf{r}'|)\rho(\mathbf{r}')\right\}\Phi_X(\mathbf{r}) = \varepsilon\Phi_X(\mathbf{r}), \tag{3}$$

respectively, where $\Psi(\mathbf{r}) = \sqrt{\rho(\mathbf{r})}$; we take $\rho_X(\mathbf{r})$ as initial guess for $\rho(\mathbf{r})$. $\mathcal{E}(\rho)$ is the density functional of Ref. [10], and $\mathcal{V}_{\text{He}-\text{He}}$ is the Aziz potential [11]. Once $\rho(\mathbf{r})$ and $\Phi_X(\mathbf{r})$ have been determined, they are used as initial conditions to solve the TD-DFT and time-dependent Schrödinger equations (TDSE) [8] with $\mathcal{V}_{\text{He}-\text{He}}$ substituted by $\mathcal{V}_{\text{He}-\text{He}^*}$. The TDSE is solved using the test particles approach [12].

Supplementary Fig. 3 shows two evolutions of the droplet-He* system complementing those presented in the main text. Animated versions for $d = 0$, 0.2, and 0.4 nm are available online. Note that when $d = 0.4$ nm, the probability density of He* splits in two, indicating that the He* atom has some probability to remain attached to the droplet, and some to be ejected. This type of scenario had provoked some discussions for the case of an electron injected into liquid He [13, 14].

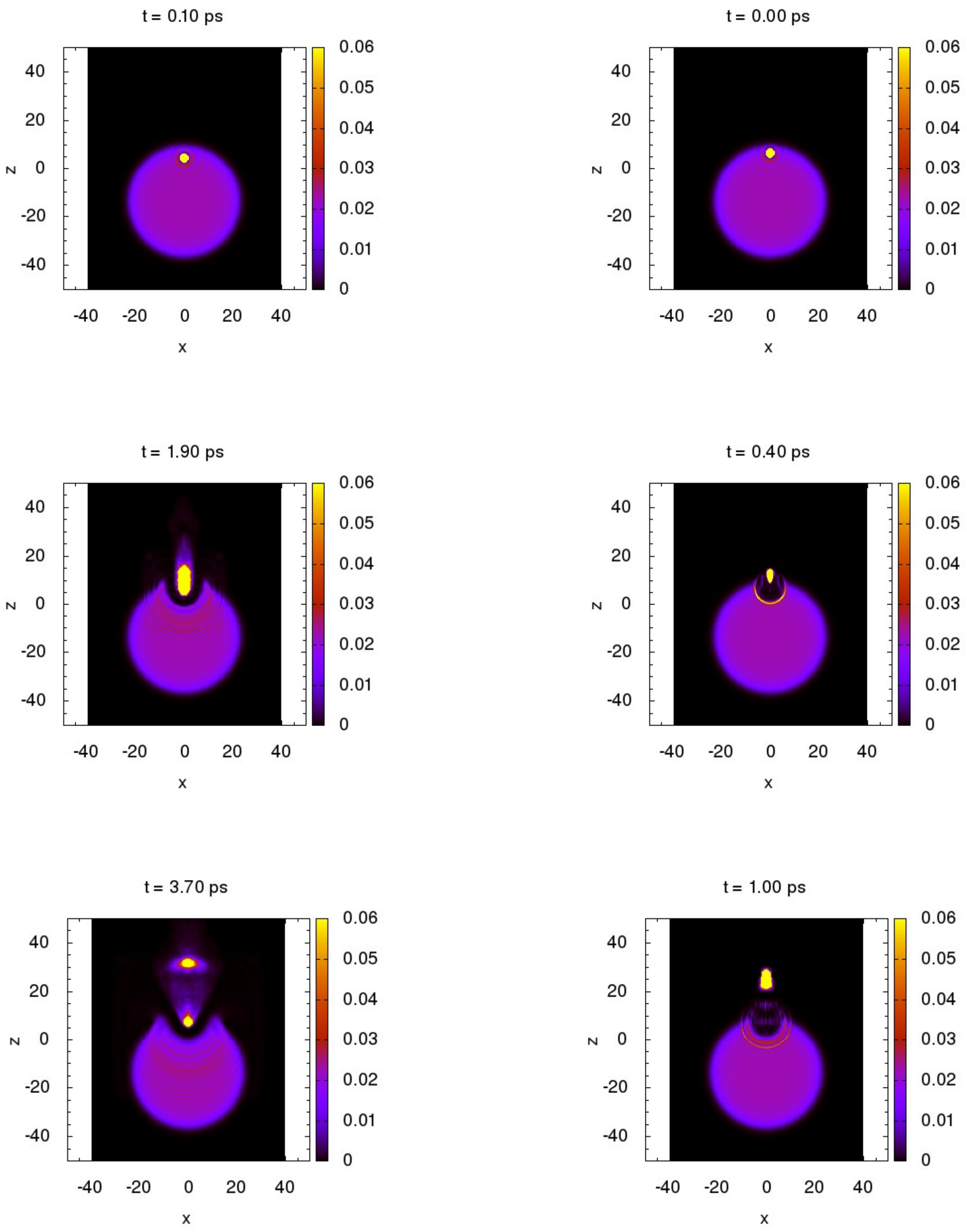

Supplementary Fig. 3: Evolution of the simulated He density distribution. The initial distance of the He* (yellow dot) from the droplet surface is 0.4 nm (left column) and 0.2 nm (right column). The He droplets contain 1000 He atoms and have a radius of 2.2 nm.

Supplementary References

[1] M. Shcherbinin, A.C. LaForge, M. Hanif, R. Richter, and M. Mudrich, "Penning Ionization of Acene Molecules by Helium Nanodroplets," J. Phys. Chem A **122**, 1855–1860 (2018).

[2] O. Kornilov, O. Bünermann, D. J. Haxton, S. R. Leone, "Femtosecond Photoelectron Imaging of Transient Electronic States and Rydberg Atom Emission from Electronically Excited He Droplets," J. Phys. Chem. A **115**, 7891–7900 (2011).

[3] H. Buchenau, J. P. Toennies, and J. A. Northby, "Excitation and ionization of ^{4}He clusters by electrons," J. Chem. Phys. **95**, 8134–8148 (1991).

[4] D. S. Peterka, J. H. Kim, C. C. Wang, L. Poisson, and D. M. Neumark, "Photoionization Dynamics of Pure Helium Droplets," J. Phys. Chem. A **111**, 7449–7459 (2007).

[5] D. Buchta, S. R. Krishnan, N. B. Brauer, M. Drabbels, P. O'Keeffe, M. Devetta, M. Di Fraia, C. Callegari, R. Richter, M. Coreno, K. C. Prince, F. Stienkemeier, J. Ullrich, R. Moshammer, and M. Mudrich, "Extreme ultraviolet ionization of pure He nanodroplets: Mass-correlated photoelectron imaging, Penning ionization, and electron energy-loss spectra," J. Chem. Phys. **139**, 084301 (2013).

[6] M. Shcherbinin, A. C LaForge, V. Sharma, M. Devetta, R. Richter, R. Moshammer, T. Pfeifer, and M. Mudrich, "Interatomic Coulombic decay in helium nanodroplets," Phys. Rev. A **96**, 013407 (2017).

[7] M. Shcherbinin, F. Vad Westergaard, M. Hanif, S. R. Krishnan, A. C. LaForge, R. Richter, T. Pfeifer, and M. Mudrich, "Inelastic scattering of photoelectrons from He nanodroplets," J. Chem. Phys. **150**, 044304 (2019).

[8] F. Ancilotto, M. Barranco, F. Coppens, J. Eloranta, N. Halberstadt, A. Hernando, D. Mateo, and M. Pi, "Density functional theory of doped superfluid liquid helium and nanodroplets," Int. Rev. Phys. Chem. **36**, 621–707 (2017).

[9] J. Boronat and J. Casulleras, "Monte Carlo analysis of an interatomic potential for He," Phys. Rev. B **49**, 8920–8930 (1994).

[10] F. Ancilotto, M. Barranco, F. Caupin, R. Mayol, and M. Pi, "Freezing of ^{4}He and its liquid-solid interface from density functional theory," Phys. Rev. B **72**, 214522 (2005).

[11] R. A. Aziz, F. R. W. McCourt, and C. C. K. Wongs, "A new determination of the ground state interatomic potential for He_2," Mol. Phys. **61**, 1487—1511 (1987).

[12] R.E. Wyatt, "Quantum dynamics with Trajectories," Springer, New York (2005).

[13] R. Jackiw, C. Rebbi, and J. R. Schrieffer, "Fractional electrons in liquid helium?," J. Low Temp. Phys. **122**, 587–589 (2001).

[14] W. Wei, Z. Xie, L. N. Cooper, G. M. Seidel, and H. J. Maris, "Study of exotic ions in superfluid helium and the possible fission of the electron wave function," J. Low Temp. Phys. **178**, 78–117 (2015).
